# Computational Modeling of the Liver Arterial Blood Flow for Microsphere Therapy: Effect of Boundary Conditions

**DOI:** 10.3390/bioengineering7030064

**Published:** 2020-06-29

**Authors:** Amirtahà Taebi, Rex M. Pillai, Bahman S. Roudsari, Catherine T. Vu, Emilie Roncali

**Affiliations:** 1 Department of Biomedical Engineering, University of California Davis, One Shields Ave., Davis, CA 95616, USA; 2 Department of Radiology, University of California Davis, 4860 Y Street, Suite 3100, Sacramento, CA 95817, USA; rexpillai@ucdavis.edu (R.M.P.); catvu@ucdavis.edu (C.T.V.); 3 Kaiser Permanente Northern California, Oakland, CA 94612, USA; roudsari@ucdavis.edu

**Keywords:** radioembolization, chemoembolization, hepatic arterial tree, multiscale modeling, computational fluid dynamics, boundary conditions

## Abstract

Transarterial embolization is a minimally invasive treatment for advanced liver cancer using microspheres loaded with a chemotherapeutic drug or radioactive yttrium-90 (^90^Y) that are injected into the hepatic arterial tree through a catheter. For personalized treatment, the microsphere distribution in the liver should be optimized through the injection volume and location. Computational fluid dynamics (CFD) simulations of the blood flow in the hepatic artery can help estimate this distribution if carefully parameterized. An important aspect is the choice of the boundary conditions imposed at the inlet and outlets of the computational domain. In this study, the effect of boundary conditions on the hepatic arterial tree hemodynamics was investigated. The outlet boundary conditions were modeled with three-element Windkessel circuits, representative of the downstream vasculature resistance. Results demonstrated that the downstream vasculature resistance affected the hepatic artery hemodynamics such as the velocity field, the pressure field and the blood flow streamline trajectories. Moreover, the number of microspheres received by the tumor significantly changed (more than 10% of the total injected microspheres) with downstream resistance variations. These findings suggest that patient-specific boundary conditions should be used in order to achieve a more accurate drug distribution estimation with CFD in transarterial embolization treatment planning.

## 1. Introduction

Transarterial embolization is an interventional radiology procedure that has been increasingly used for unresectable liver cancer treatment [1]. The treatment consists of injecting microspheres loaded with chemotherapeutic agents or radioactive yttrium-90 (^90^Y) with a catheter through the hepatic artery to target tumors in the liver. Recent studies have shown the potential of computational fluid dynamics (CFD) in tracking the embolization microspheres [2,3,4]. Simulation of the blood flow behavior inside the hepatic arterial tree can help predict the microsphere trajectories and thus their distribution between different liver segments. Using these predictions, the injection location and volume can be optimized before the treatment to maximize the tumor targeting. The CFD simulation results are however affected by different factors that need to be carefully studied. Among these parameters, the boundary conditions imposed on the computational domain play an important role [5]. Although the effect of boundary conditions on the lower-dimensional models (e.g., 0D and 1D model) of the hepatic artery has been discussed in the literature [6,7], it has not been quantitatively investigated for 3D CFD simulation of the hepatic arterial tree hemodynamics. Three-dimensional CFD simulation provides fine details of the flow internally within the 3D domain. Thus, it can show how boundary condition variations change the local field parameters such as velocity field and flow streamlines inside the hepatic arterial tree.

These boundary conditions can vary due to different physiological conditions and factors, which should be considered to maximize simulation accuracy in the prediction of the chemotherapeutic drug or ^90^Y microsphere distribution. In transarterial embolization, the balloon occlusion of the hepatic artery affects the hepatic arterial tree hemodynamics [8]. The presence of the catheter also changes the flow field characteristics such as downstream blood pressure and velocity [9]. In addition, as tumors grow in the liver, the hepatic vascular structure around the lesion changes, which in turn leads to modified hepatic artery hemodynamics compared to a normal liver. This vascular structure modification can affect the blood flow rate into different liver segments [10] which may result in a different delivery rate of the therapeutic microspheres to the tumor and surrounding healthy parenchyma. The hepatic arterial buffer response is another feature of the liver dual blood supply system that enables the hepatic artery to compensate for the flow changes in the portal vein system. Hence, hepatic arterial tree blood flow and pressure can change due to this feature [11]. Other conditions such as increased intra-abdominal pressure and acute cholangitis may also lead to hepatic artery blood flow and pressure variations [12,13]. All these conditions alter the hepatic arterial tree hemodynamics and may thus change the drug distribution in the liver.

Radioembolization is promising for intermediate and advanced-stage disease thanks to its microembolic effect (due to smaller microspheres than chemotherapy beads) and localized targeting of the tumors with radiation. Its current clinical practice however requires extra steps such as extensive treatment planning where a multidisciplinary team of interventional radiologists and nuclear medicine physicians decide the ^90^Y activity to inject and the injection location. The current methods used to determine the injection activity and location are based on simplified assumptions such as the homogeneous distribution of the microspheres in the liver [14]. Any improvements in these methods would result in better treatment planning. This in turn would enhance the overall treatment success in terms of maximizing the number of microspheres delivered to the tumor while minimizing the delivery to the surrounding healthy liver. We have developed a new dosimetry called CFDose that utilizes CFD to predict the nonhomogeneous distribution of ^90^Y microspheres [15]. Since this method is based on CFD simulations of the hepatic blood flow, the CFD boundary conditions can affect its accuracy and must be chosen carefully. To address this concern, the first step is to characterize the sensitivity of the microsphere distribution to the outlet conditions in a CFD simulation. In this paper, the effect of outlet pressure variations on the estimation of the microsphere distribution in the liver with CFDose was studied by changing the resistance of the downstream vasculature. Future studies should compare the results of this study against the real distribution of the microspheres after the treatment (e.g., using PET scans). This helps determine the most appropriate boundary conditions in our CFD studies and understand how the tumor presence changes the arterial tree downstream conditions.

## 2. Materials and Methods

The effect of different boundary conditions on the hepatic arterial tree hemodynamics was investigated in this study. For this purpose, we segmented the hepatic arterial tree of a patient with hepatocellular carcinoma in the right liver lobe from the standard-of-care cone-beam computed tomography (CBCT) scan. CFD simulations were then conducted for different outlet boundary conditions. In this section, the 3D model preparation, mesh generation, and CFD simulation details are presented. The data collection and image processing were done in a single site (UC Davis Health, Sacramento, CA). The prospective study approved by the UC Davis Institutional Review Board (IRB).

### 2.1. Computational Domain Extracted from CBCT

**Figure 1:**
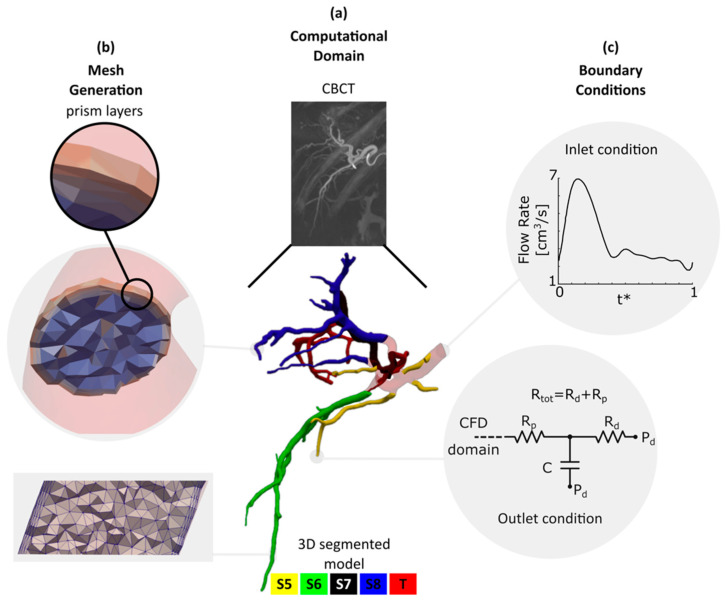
Computational domain, mesh generation and boundary conditions. (**a**) Computational domain: Cone-beam CT scan and the 3D segmented hepatic arterial tree color-coded according to Couinaud system. S5, S6, S7, S8 and T stand for liver segments 5, 6, 7, 8 and tumor, respectively; (**b**) mesh generation: mesh details and prism layers employed to resolve the governing equations in the boundary layers; (**c**) boundary conditions: inlet flow rate and the 3-element Windkessel circuit imposed at each outlet. A total of 23 simulations with different *R_tot_* and *R_d_*/*R_p_* were carried out.

A radio-opaque contrast (Omnipaque 300) was administered in the hepatic arterial tree immediately before the CBCT scan. The scan was done under breath-hold using a Siemens Artis Zeego angiography system. The scan was then rendered as a 3D volume of 185 1024 × 1024 images with a thickness of 1 mm and an in-plane pixel size of 0.25 × 0.25 mm^2^ using Siemens DynaCT software. Further preprocessing and inspections, detailed in [16], were done in MATLAB (R2018b, The MathWorks, Inc., Natick, MA, USA). The right liver segments were annotated from S5 to S8 according to the Couinaud system [17] by an interventional radiologist. The tumor was mainly located in segment 7 (S7). The arteries supplying blood to each liver segment and the tumor are color-coded in Figure 1a.

A marching cubes algorithm was then employed to determine the location of the hepatic arterial tree wall. Due to low image resolution and contrast, the extracted surfaces required additional smoothing before they could be used as the 3D CFD model. They were thus first smoothed with Taubin’s algorithm. The centerline and maximum inscribed radius (*r_ins_*) of the arteries were calculated from the Voronoi diagram. A moving average filter was then used to smooth them. It is legitimate to assume that the arteries in the hepatic arterial tree have a circular cross-section [18,19]. Thus, a circular surface centered at the centerline with a radius of smoothed *r_ins_* was created at each point along the centerline trajectory to build the CFD model (Figure 1a). The final hepatic artery model had an inlet diameter of 4.6 mm at the right hepatic artery (RHA). Our segmentation method was able to detect arteries with a diameter (*d*) as small as 0.45 mm. Open source vascular modeling toolkit vmtk (www.vmtk.org), ParaView (www.paraview.org) and OpenFlipper (www.openflipper.org) were used to create, smooth, and inspect the 3D model. The 3D model was finally saved in stereolithography format as a surface mesh.

### 2.2. Meshing

A tetrahedral mesh with regional refinement was generated using TetGen [20], an open-source package for mesh generation included in SimVascular (www.simvascular.org). The mesh size was selected based on a mesh independency study where the discretization uncertainty, excluding the modeling error, was calculated for three different mesh sizes with a refinement factor of 2.29 according to the ASME recommendations for CFD studies [21]. The details of the mesh independency study is described in [22]. Based on the mesh independency study, the computational model was meshed with a total number of 8.8-M linear tetrahedral elements with a maximum edge size of 0.18 mm. The velocity and pressure profiles have a steep gradient in the boundary layers near the arterial walls. Therefore, three prism layers were employed to resolve the flow field in this region. The first prism layer (i.e., the closest to the artery’s center) thickness was half that of the adjacent mesh element. The thickness of the following layers was decreased by 20%. The mesh details in two sample cross-sections are shown in Figure 1b.

### 2.3. Governing Equations

Three-dimensional Navier–Stokes conservation of mass and momentum equations for laminar incompressible flow were solved to calculate the flow field in the computational domain:

(1) ∇.(ρu)=0,

(2) $$\rho (\frac{\partial u}{\partial t}+u.\nabla u)=-\nabla p+\mu \nabla^{2}u+\frac{1}{3}\mu \nabla \left(\nabla .u\right),$$

where *u*, *p*, *ρ* and *μ* are the flow velocity, flow pressure, blood density and dynamic viscosity, respectively. In large arteries such as the hepatic artery, the blood flow behavior can be studied using a Newtonian model [23]. Therefore, we assumed that blood was incompressible and Newtonian with a density and dynamic viscosity of 1.06 gr/cm^3^ and 0.04 gr/cm·s, respectively. To examine the assumption of laminar flow, the Reynolds number was calculated and was found to be well below the laminar–turbulent transition in all cases (described in Section 2.4).

### 2.4. Boundary Conditions

Since the tumor (T) was mainly located in the right lobe, the left lobe was not included in the computational domain. The computational model thus consisted only of the hepatic arterial tree in the right lobe with an inlet at the RHA. The presence of the tumor affects the local vascular structure as well as the blood flow rate to the liver tissue with the tumor. Previous studies suggested that the liver lobe containing a tumor receives 1.45 times more blood flow than a healthy liver lobe [24]. Thus, for the inlet flow rate, a representative pulsatile waveform (Figure 1c) with a parabolic velocity profile was approximated and modified to account for the tumor effect based on published data [24,25]. The average flow rate at the inlet was 3.5 cm^3^/s. Here, t* is the time vector nondimensionalized by the cardiac cycle duration.

The arterial tree included in this study had 46 outlets with the following characteristics; S5:6 outlets with 0.81 < *d* < 1.84 mm, S6:10 outlets with 0.45 < *d* < 1.35 mm, S7:17 outlets with 0.59 < *d* < 1.59 mm, S8:13 outlets with 0.6 < *d* < 1.55 mm. This makes it difficult to measure the in vivo static pressure and flow rate at the outlets. There are different methods to account for the effect of the downstream vascular bed on the upstream computational domain [26]. In this study, the downstream vasculature of each outlet was modeled with a 3-element Windkessel-lumped parameter network (LPN) as shown in Figure 1c. The LPN model at each outlet consisted of a proximal resistance (*R_p_*), compliance (*C*), and distal resistance (*R_d_*) that was connected to a distal pressure (*P_d_*) [27]. The distal pressure was assumed to be constant and determined from a separate closed LPN model of the total body [28], which was tuned using the patient’s heart rate, systolic and diastolic pressure (72 bpm, 80 mmHg and 110 mmHg, respectively). In all the simulations, *P_d_* was 15 mmHg. The 3-element Windkessel LPNs were coupled with the 3D domain to iteratively pass the flow and pressure information between the 0D and 3D domains as described in [29]. *R_tot_* and *C_tot_* are the total resistance and compliance imposed at the outlets feeding each liver segment, respectively. For the healthy segments S5, S6 and S8, the *R_tot_* and *C_tot_* values were adjusted such that the blood pressure dropped by 4.5–9 mmHg from the RHA to the outlets during a cardiac cycle based on [30]. A *C_tot_* of 8.93 × 10^−7^ cm^5^/dyne was used for each liver segment. This compliance was split between the outlets ending up to each liver segment using [31]:

(3) $$C_{i}=\frac{A_{i}}{\sum^{\text{​}}A_{i}}C_{tot},$$

where *C_i_* and *A_i_* are the compliance and cross-sectional area of the *i^th^* outlet of a specific liver segment. *R_tot_* values for S5, S6 and S8 were fixed to 9.1 × 10^4^, 14.6 × 10^4^ and 4.4 × 10^4^ dyne·s/cm^5^, respectively, for all simulations. To study the effect of outlet resistance on the hepatic artery hemodynamics and flow distribution, the *R_tot_* of S7, which included the tumor feeding branches, was varied from ~4 to 8 × 10^4^ dyne·s/cm^5^. Each segment’s total resistance was then split between the outlets feeding that segment as follows:

(4) $$R_{i}=\frac{\sum^{\text{​}}A_{i}}{A_{i}}R_{tot},$$

where *R_i_* is the total resistance (*R_d_* + *R_p_*) at the *i^th^* outlet of that segment. Due to a lack of information about the *R_d_*/*R_p_* ratio in the hepatic arterioles, we performed the simulations by varying *R_d_*/*R_p_* from 1 to 10 (at fixed *R_tot_* values). This let us also investigate the effect of distal and proximal resistance variations on the hepatic flow distribution and outlet pressure. The choice of 1 < *R_d_*/*R_p_* < 10 was based on the fact that the distal resistance (i.e., microvasculature) is typically much larger than the proximal resistance (arteries). In addition, the role of *R_d_* in improving the high-frequency response of the model could be studied by varying *R_d_*/*R_p_*. A complete list of *R_tot_* and *R_d_*/*R_p_* used is provided in Table 1. All simulation cases used a rigid wall assumption with a no-penetration condition and no-slip condition along them.

### 2.5. Solver

The governing equations were solved using a finite element method in the open-source software SimVascular [32]. The solver was validated for arterial blood flow simulation in many previous studies including [33,34,35]. The equations were discretized and advanced in time by the Galerkin and a second–order accurate generalized-alpha method (which was unconditionally stable), respectively [36,37]. The solution was stabilized by the streamwise upwind Petrov–Galerkin method. A time step of 0.3 ms was selected such that the Courant–Friedrichs–Lewy (CFL) number was about 1. The simulations were run for five cardiac cycles until periodically stable flow and pressure states were reached. The conservation of mass between the inlet and the outlet was also verified.

### 2.6. Postprocessing of CFD Results

ParaView was used for the postprocessing of the simulation results obtained from the last cardiac cycle. We extracted velocity, flow rate, and pressure at different arterial segments throughout the hepatic arterial tree model. The pressure at each arterial outlet, *P*(*t*), was calculated using Equation (5) [27].

(5) $$P\left(t\right)=\left(P_{0}-R_{p}Q_{0}-P_{d}\right)e^{\frac{-t}{R_{d}C}}+P_{d}+R_{p}Q+\int_{0}^{t}\frac{e^{\frac{-\left(t-\tau \right)}{R_{d}C}}}{C}Q\left(\tau \right)d\tau ,$$

where *Q* is the outlet flow rate and *P*_0_ and *Q*_0_ are the pressure and flow rate at t = 0. The average pressure (*P_avg_*) and flow rate (*Q_avg_*) at each outlet were also calculated. The time lag in the outlet pressure due to *R_d_*/*R_p_* variations was also calculated at each outlet. The dimensionless time lag between the *R_d_*/*R_p_* = 1 and *R_d_*/*R_p_* = 3, 5 and 7 was called t_13_, t_15_ and t_17_, respectively.

As described earlier, the main goal of this study was to evaluate the effect of boundary conditions on liver arterial blood flow distribution. Investigating local field variations such as flow streamlines can shed light on how blood flow is distributed between the arterial branches under different outlet boundary conditions. Particle release maps (PRMs) indicate the final destination (i.e., exit outlet) of a streamline that originally passed through the inlet cross-section. PRMs are therefore a powerful way to investigate the effect of outlet boundary conditions on the blood flow distribution from the inlet to the outlets. PRMs can also help predict the therapeutic microsphere distribution when Stokes number is less than one. To calculate the PRMs, a total of 107,000 seed points was selected at the inlet for each case and the streamlines were integrated from these seed points to the outlet position they reached. The seed points that reached the same liver segment were labeled with the same color to mark the inlet regions that supplied blood to that segment. To study the effect of boundary conditions on the PRMs, they were calculated and plotted at 10 equal intervals of the cardiac cycle.

This study is part of our efforts to develop CFDose, a CFD-based dosimetry tool for liver cancer radioembolization. The absorbed dose to tissue is directly dependent on the number of microspheres accumulating in the arterioles, which motivated this work to also estimate the ^90^Y microsphere delivery to different liver segments. The Stokes number (*St*) was calculated using the blood and ^90^Y glass microsphere properties (*ρ_m_* = 3600 kg/m^3^, *d_m_* = 20–30 µm [5]). Since *St* << 1, we assumed that the microspheres follow the blood flow streamlines. Therefore, the number of microspheres reached to each liver segment was correlated to the cumulative blood flow to that segment (i.e., summation of blood flow to the arterial outlets feeding each liver segment).

## 3. Results

### 3.1. Hepatic Arterial Tree Hemodynamics

**Figure 2:**
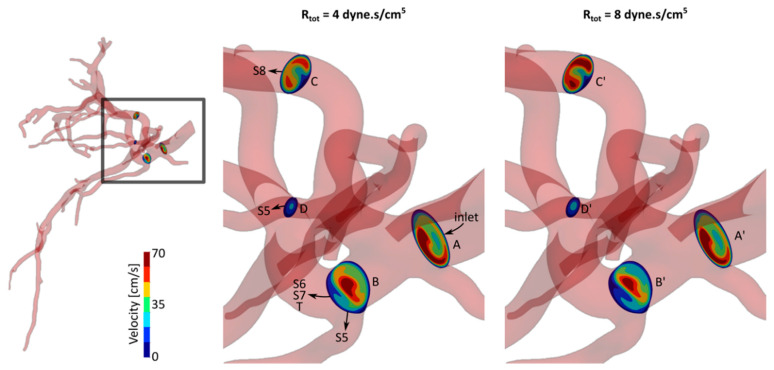
Axial velocity profiles for two cases with *R_d_*/*R_p_* = 1 and different total resistance at S7 outlets, *R_tot_* = 4 and 8 × 10^4^ dyne·s/cm^5^.

We investigated the effect of the downstream resistance variations of S7 (including tumor) on the hepatic arterial tree hemodynamics. Figure 2 shows the comparison between the axial velocity profiles of S7 *R_tot_* = 4 and 8 × 10^4^ dyne·s/cm^5^ (A–D and A’–D’, respectively) with *R_d_*/*R_p_* = 1 at four selected cross-sections. These profiles are shown at the maximum inlet flow rate instant (t* = 0.15). The cross-sections were selected on the upstream side of the computational domain such that they include blood flow delivered to different liver segments. Sections A and A’ were selected near the arterial tree inlet. Sections B and B’ were right before a bifurcation where one of the daughter branches delivered blood to part of S5 and the second one fed S6, S7 and T. Sections C and C’ were on the main branch supplying S8. Finally, sections D and D’ were on another branch feeding S5. See supplementary material for a video that shows the velocity variations during a cardiac cycle.

The axial velocity profile and magnitude were similar with small variations between sections A and A’. The velocity magnitude in section B was larger than in section B’. This can be seen by comparing the regions color-coded with red and yellow in both sections. It is worth noting that the entire blood supply of S7 passed from sections B and B’. The larger velocity at section B can be therefore due to the smaller S7 resistance of this case (i.e., *R_tot_* = 4 × 10^4^ dyne·s/cm^5^) compared to the other case. On the other hand, it can be seen that the velocity magnitude was larger at sections C’ and D’ compared to sections C and D, respectively. These sections were on the way of S8 and parts of S5 which had fixed resistance between the two simulation cases shown in Figure 2. Therefore, by increasing the resistance at S7 outlets, the blood outflow increased at other segment outlets. The velocity profiles changed similarly for other *R_tot_* values from 4 to 8 × 10^4^ dyne·s/cm^5^. Velocity profiles with smaller changes were observed for other *R_d_*/*R_p_* ratios.

### 3.2. Particle Release Maps

**Figure 3:**
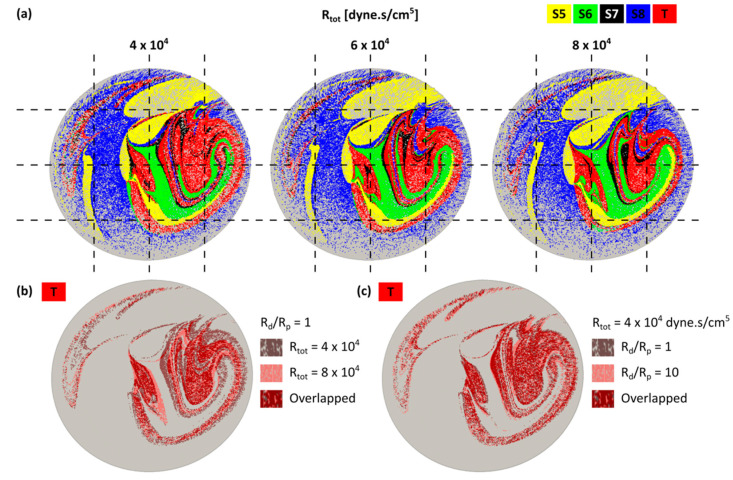
(**a**) Particle release maps (PRMs) at the inlet of the hepatic arterial tree for *R_tot_* = 4, 6, 8 × 10^4^ dyne·s/cm^5^ with *R_d_*/*R_p_* = 1. Streamlines delivered to the same liver segment are color-coded; (**b**) comparison between the tumor PRMs for *R_tot_* = 4 and 8 × 10^4^ dyne·s/cm^5^ with *R_d_*/*R_p_* = 1; (**c**) comparison between the tumor PRMs for *R_d_*/*R_p_* = 1 and 10 (*R_tot_* = 4 × 10^4^ dyne·s/cm^5^). In both (**b**) and (**c**), overlap between the PRMs are shown in dark red.

The PRMs of 10 equal intervals of the cardiac cycle were produced for all cases. Results showed a transient topology during the cardiac cycle. To compare the effect of downstream vasculature resistance on the particle trajectories, the PRMs were studied for all cases listed in Table 1. Three representative cases with *R_tot_* = 4, 6 and 8 × 10^4^ dyne·s/cm^5^ and *R_d_*/*R_p_* = 1 at t* = 0.15 are shown in Figure 3a. Streamlines are color-coded according to the liver segment they supplied. Changes in the downstream resistance (*R_tot_*) affected the streamline trajectories, as illustrated by the differences in PRMs for different *R_tot_* or *R_d_*/*R_p_* ratios.

Figure 3b is a subset of Figure 3a only showing the tumor PRMs for two different *R_tot_*. Figure 3c shows the tumor PRMs for two different *R_d_*/*R_p_*. In each figure, overlapping areas of the two PRMs are shown in dark red. The larger overlap area in Figure 3b than Figure 3c indicates that PRMs changed more with *R_tot_* than with *R_d_*/*R_p_*. This can be due to local field changes shown in the previous section or alterations of the streamline trajectories.

### 3.3. Effect of R_tot_ and R_d_/R_p_ Ratio on Outlet Pressure and Flow Rate

**Figure 4:**
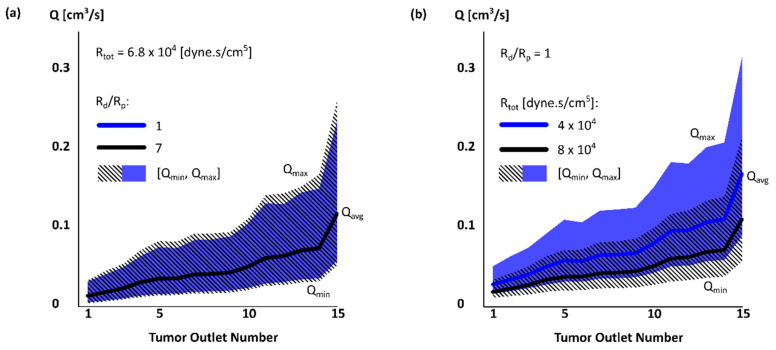
Flow rate *Q_avg_* [*Q_min_*, *Q_max_*] in tumor outlets for two simulation cases with (**a**) fixed *R_tot_* and varying *R_d_*/*R_p_* and (**b**) fixed *R_d_*/*R_p_* and varying *R_tot_*.

The effect of outlet diameter on outlet pressure and flow rate is shown in Appendix A. Figure 4 shows the flow rate at tumor outlets with (a) a fixed *R_tot_* and varying *R_d_*/*R_p_* and (b) fixed *R_d_*/*R_p_* and varying *R_tot_*. Varying *R_d_*/*R_p_* did not significantly change *Q_avg_* (~1.2%), but slightly affected the range of flow rate (~13% when *R_d_*/*R_p_* increased from 1 to 7). The flow rate extrema had a similar trend to *Q_avg_*. The range of flow rate changed similarly at other tumor outlets with *R_d_*/*R_p_*. In contrast, *Q_avg_* was significantly affected by *R_tot_*. In tumor outlets, *Q_avg_* decreased by ~36% with increasing *R_tot_* (Figure 4b) while increased in the other liver segment outlets by ~22% (not shown). In addition, the flow rate range reduced by 34% when *R_tot_* increased.

Although *R_tot_* had a larger effect on the absolute values of the flow rate *Q*, *R_d_*/*R_p_* had a larger effect on the flow rate range relative to *Q_avg_*. The range changed from [0.51, 2.01] *Q_avg_* to [0.46, 2.17] *Q_avg_* with *R_d_*/*R_p_*. Varying *R_tot_* only changed the range from [0.52, 1.95] *Q_avg_* to [0.5, 1.99] *Q_avg_*. The outlet pressure was similarly more affected by *R_tot_* than *R_d_*/*R_p_* (13% vs only 0.1%). The *P_min_*/*P_avg_* and *P_max_*/*P_avg_* were slightly changed by *R_tot_* (~3%). *P_avg_* also increased by *R_tot_* at other liver segments, but with a smaller increase rate than S7 (including T).

**Figure 5:**
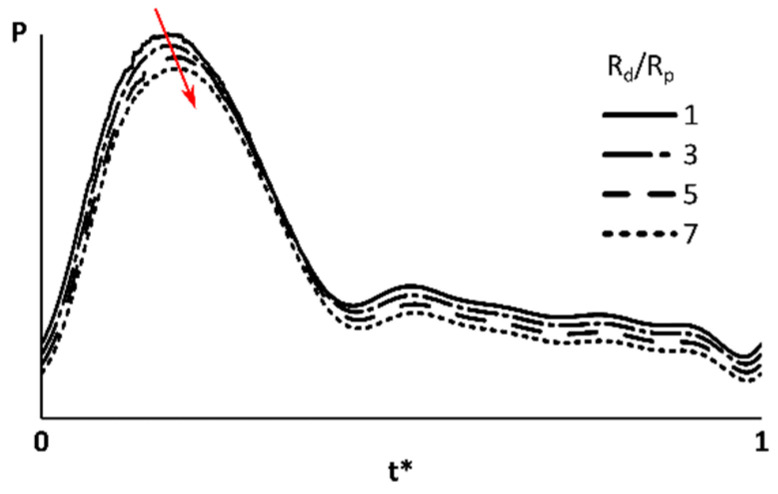
Pressure at an outlet feeding the tumor. The S7 (which includes tumor) total resistance was 6.8 × 10^4^ dyne·s/cm^5^. Red arrow shows how the peak pressure time shifted by increasing *R_d_*/*R_p_*. The pressure waveforms moved in vertical direction for a better illustration.

Figure 5 shows the pressure waveforms for different *R_d_*/*R_p_* ratios (*R_tot_* = 6.8 × 10^4^ dyne·s/cm^5^) at tumor outlet #1 (*d* = 0.59 mm). Results showed that increasing *R_d_*/*R_p_* created a time lag in the pressure waveform in this outlet. A similar time lag was observed in the other liver segments (Table 2). At S5, S6 and S8 outlets, the flow waveforms with an *R_d_*/*R_p_* greater than 1 had a time lag. In contrast, S7 outlets showed a time lead for the cases with larger *R_d_*/*R_p_*. The pressure variations are partly due to the flow rate changes (Equation (5)). In addition, the time constant in Equation (5), *R_d_C* determines how fast the outlet pressure responds to the flow rate variations and therefore could cause the time lag if changed with *R_d_*/*R_p_*.

### 3.4. Blood Flow Distribution and ^90^Y Delivery in Liver

**Figure 6:**
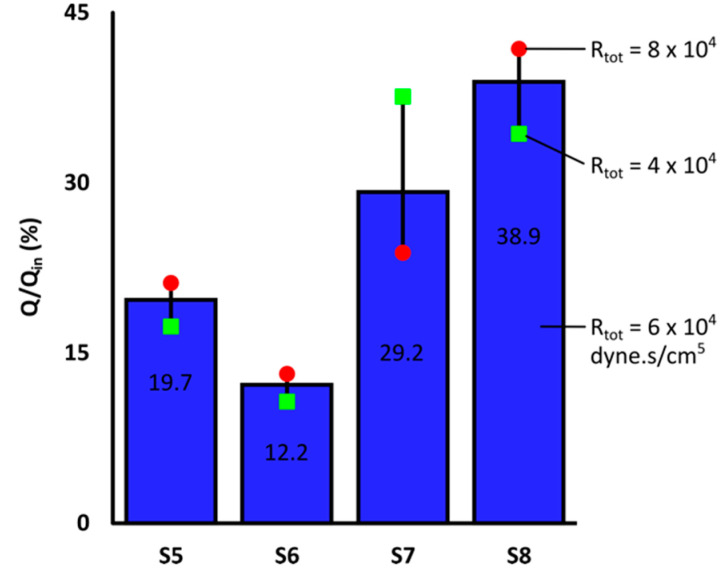
Blood flow distribution between right liver segments for *R_d_*/*R_p_* = 1 with S7 *R_tot_* = 6 × 10^4^ dyne·s/cm^5^. S7 included blood flow to the tumor. Black lines show the blood flow fluctuations due to S7 *R_tot_* variations in the range of [4,8] × 10^4^ dyne·s/cm^5^.

Figure 6 shows the cumulative blood flow as a percentage of the total blood flow. The black lines represent the blood flow fluctuations in each segment when S7 *R_tot_* varied between 4–8 × 10^4^ dyne·s/cm^5^. The relationship between the blood flow (*Q*/*Q_in_*) and *R_tot_* variations in each segment could be better described with a second-order polynomial (R^2^ > 0.999) rather than a linear (R^2^ ~0.98) or exponential (R^2^ ~0.97) fit. While *Q*/*Q_in_* to S7 decreased from 38% to 24% by increasing S7 *R_tot_* from 4 to 8 × 10^4^ dyne·s/cm^5^, it increased by 4%, 2% and 8% in S5, S6 and S8, respectively (reversed trends indicated by green and red markers). These results indicated that a resistance decrease in S7 could result in blood flow reversal in other branches. In addition, the local changes in the blood velocity and pressure fields as well as streamline trajectories affected the blood flow distribution between different liver segments. Consequently, the number of ^90^Y microspheres received by the liver segments changed, since they are assumed to closely follow the blood flow streamlines (Section 2.6). Results for other *R_d_*/*R_p_* ratios were similar.

## 4. Discussion

This is the first 3D CFD study of the hepatic arterial tree aiming at quantifying the effect of downstream boundary conditions on the arterial tree hemodynamics in a realistic image-extracted computational domain with 46 outlets. Previous studies [6,7] had analyzed the effect of boundary conditions in lower-dimensional models such as 0D and 1D models. However, these models are high-level abstractions of the hepatic arterial blood flow and cannot reveal the effect of boundary conditions on the fine details of the flow such as velocity field, streamline and particle trajectory variations. In this study, the effect of outlet boundary condition was evaluated using two parameters of the 3-element Windkessel model: the total resistance *R_tot_* and the ratio between distal and proximal resistances *R_d_*/*R_p_* (Figure 1). The RCR values were selected such that blood flow and pressure conditions between the inlet and outlets were representatives of realistic physiological conditions. We assumed that the tumor presence would only affect the downstream resistance of the tumor-feeding branches, which should be validated in future studies. Therefore, the impedance of the other branches remained unchanged. Varying the resistance of one liver segment at a time also changed the resistance ratio between different liver segments. This in turn let us draw a conclusion with a fewer number of CFD simulations.

Results demonstrated that the downstream vasculature resistance affected the local flow fields such as the velocity and pressure in the hepatic arterial tree. The blood flow streamline trajectories also changed with *R_tot_* or *R_d_*/*R_p_*, which resulted in different PRMs. Among the two parameters analyzed in this study, *R_tot_* had a higher impact on the hemodynamics. It significantly changed the outlet flow rate and pressure as well as the streamline trajectories and PRMs. *R_d_*/*R_p_* ratio had a negligible effect on the outlet flow rates and limited effect on the local fields and PRMs. Changes in the microsphere distribution in turn changed the ^90^Y activity distribution and ultimately affected the absorbed dose in the tumor and healthy liver. Thus, *R_tot_* and in general the outlet boundary condition is one of the key drivers of blood flow in the computational domain. This means that *R_tot_* should be chosen (or measured) carefully in the CFD simulation of the hepatic arterial tree applied to drug delivery, chemoembolization and radioembolization. For instance, *R_tot_* can significantly change the dosimetry estimation in CFDose [15] which is calculated based on the CFD analysis of the hepatic blood flow. This finding is consistent with a previous qualitative review study [5] that suggested boundary conditions affect the outcome of blood flow simulation in the hepatic arterial tree.

However, some hemodynamic parameters such as outflow static pressure and flow rate could not be measured invasively or non-invasively in patients. This constitutes the motivation of the future works to conduct the development of sophisticated simulations that can ultimately be compatible with clinical patient care. In this study, the effect of the distal vasculature was approximated using 3-element Windkessel models which were coupled to the 3D geometry outlets. The size of the segmented distal vessels were limited to the CBCT image resolution. Higher resolution and contrast-to-noise images can lead to segmentation of smaller vessels. A sensitivity analysis can shed light on the effect of size of the segmented distal vasculature on the hepatic blood flow distribution.

In this study, the blood was assumed to behave like a Newtonian fluid because most of the vasculature included in the computational model had a diameter greater than one millimeter [38]. This assumption was consistent with previous studies investigating the blood flow in the hepatic arterial tree [25,39,40]. However, it is well known that blood is a non-Newtonian fluid when it circulates in smaller arteries. The non-Newtonian behavior of blood alters the primary and secondary flow patterns as well as wall shear stress, flow recirculation and vortices in the bloodstream [41]. These changes can significantly affect the flow distribution in the hepatic arterial tree and therefore should be addressed in future studies especially if smaller vasculature is included in the computational model.

## 5. Conclusions

The effect of downstream vasculature resistance on the 3D hemodynamics of the hepatic arterial tree of a patient with hepatocellular carcinoma was investigated quantitatively for the first time to our knowledge. This sensitivity analysis helps understand how the results of the CFD studies of the liver blood flow depend on the outlet boundary conditions. In the context of ^90^Y microsphere dosimetry such as implemented in CFDose, results showed that the total resistance of the liver segments changed the outlet pressure and flow rate, which would consequently affect the blood flow rate and the number of microspheres delivered to each liver segment. Thus, to achieve more accurate radioembolization dosimetry using CFDose, it is essential to carefully select the CFD boundary conditions for each patient.

## Figures and Tables

**Table 1:** *R_tot_* and *R_d_*/*R_p_* used in the computational fluid dynamics (CFD) simulations.

		*R_d_*/*R_p_*				
***R_tot_*** **[×10^4^ dyne·s/cm^5^]**	4.0	1	3	–	–	10
	4.7	1	3	–	–	10
	5.3	1	3	–	–	10
	6.0	1	3	–	–	10
	6.8	1	3	5	7	10
	7.3	1	3	–	–	10
	8.0	1	3	–	–	10

**Table 2:** The pressure waveform time lag (mean ± SD) between the cases with *R_d_*/*R_p_* = 1 and 3 (t_13_), 1 and 5 (t_15_), and 1 and 7 (t_17_).

	t_13_ [×10^−3^]	t_15_ [×10^−3^]	t_17_ [×10^−3^]
**S5**	1.5 ± 1.64	7.5 ± 1.64	12.5 ± 2.26
**S6**	0.3 ± 1.70	8.7 ± 0.95	12.3 ± 2.21
**S7**	1.4 ± 1.54	9.7 ± 1.69	14.5 ± 1.59
**S8**	0.9 ± 1.44	8.8 ± 0.83	13.1 ± 1.95

**Figure A1:**
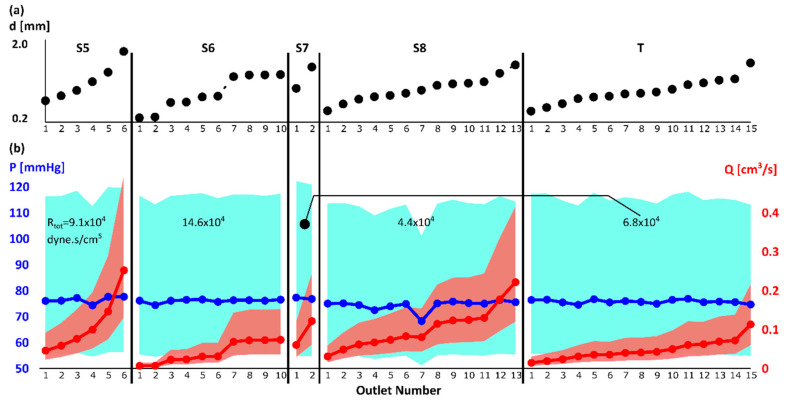
(**a**) Outlet diameter: The outlets to each liver segment were numbered with increasing diameter; (**b**) outlet average pressure *P_avg_* (blue solid line) and flow rate *Q_avg_* (red solid line). The blue and red shadows show the range [*P_min_*, *P_max_*] and [*Q_min_*, *Q_max_*], respectively. For the results shown in this figure, the *R_tot_* was 6.8 × 10^4^ dyne·s/cm^5^ and *R_d_*/*R_p_* was 1 for S7 (which included T).

## Supplementary Materials

The following are available online at https://www.mdpi.com/2306-5354/7/3/64/s1, Video S1: Axial velocity profiles for two cases with *R_d_*/*R_p_* = 1 and different total resistance at S7 outlets, *R_tot_* = 4 and 8 × 10^4^ dyne·s/cm^5^ during a cardiac cycle.

## Appendix A

For each segment, the outlets were numbered by increasing diameter (Figure A1a). The corresponding outlet pressure *P* (mmHg) and flow rate *Q* (cm^3^/s) for one of the simulations (*R_tot_* = 6.8 × 10^4^ dyne·s/cm^5^ and *R_d_*/*R_p_* = 1) are shown in Figure A1b. The results were analyzed similarly for the rest of the simulations (not shown here). The solid lines show *P_avg_* and *Q_avg_* while shadows represent the range of *P* and *Q* at each outlet. Within each liver segment, the outlet flow rate was proportional to the outlet cross-sectional area. This was expected since each liver segment resistance was split between the arterial outlets using Equation (4). The flow rate at two outlets with a similar cross-sectional area, but in different liver segments was not however similar (e.g., S8 and T outlets #4).

The outlet pressure did not present a relationship with the outlet diameter (Solid blue line in Figure A1). The average pressure (*P_avg_* ± SD) did not change with the diameter and was 77.7 ± 1.5 mmHg. For other cases not shown here, *P* and *Q* showed similar trends with a fixed *R_d_*/*R_p_* ratio and varying *R_tot_*.
